# *Rhizobium leguminosarum* Glutathione Peroxidase Is Essential for Oxidative Stress Resistance and Efficient Nodulation

**DOI:** 10.3389/fmicb.2021.627562

**Published:** 2021-02-09

**Authors:** Aiqi Hu, Xiaohong Chen, Sha Luo, Qian Zou, Jing Xie, Donglan He, Xiaohua Li, Guojun Cheng

**Affiliations:** Hubei Provincial Engineering and Technology Research Center for Resources and Utilization of Microbiology, College of Life Sciences, South-Central University for Nationalities, Wuhan, China

**Keywords:** *Rhizobium leguminosarum*, glutathione peroxidase, antioxidant function, symbiotic nitrogen fixation, quantitative proteomics

## Abstract

Glutathione (GSH) plays a key role in regulating the cellular Redox Homeostasis, and appears to be essential for initiation and development of root nodules. Glutathione peroxidase (Gpx) catalyzes the reduction of H_2_O_2_ and organic hydroperoxides by oxidation of GSH to oxidized GSH (GSSG), which in turn is reduced by glutathione reductase (GR). However, it has not been determined whether the *Rhizobium leguminosarum* Gpx or GR is required during symbiotic interactions with pea. To characterize the role of glutathione-dependent enzymes in the symbiotic process, single and double mutants were made in *gpxA* (encoding glutathione peroxidase) and *gshR* (encoding glutathione reductase) genes. All the mutations did not affect the rhizobial growth, but they increased the sensitivity of *R. leguminosarum* strains to H_2_O_2_. Mutant in GpxA had no effect on intracellular GSH levels, but can increase the expression of the catalase genes. The *gshR* mutant can induce the formation of normal nodules, while the *gpxA* single and double mutants exhibited a nodulation phenotype coupled to more than 50% reduction in the nitrogen fixation capacity, these defects in nodulation were characterized by the formation of ineffective nodules. In addition, the *gpxA* and *gshR* double mutant was severely impaired in rhizosphere colonization and competition. Quantitative proteomics using the TMT labeling method was applied to study the differential expression of proteins in bacteroids isolated from pea root nodules. A total of 27 differentially expressed proteins were identified in these root bacteroids including twenty down-regulated and seven up-regulated proteins. By sorting the down-regulated proteins, eight are transporter proteins, seven are dehydrogenase, deoxygenase, oxidase, and hydrolase. Moreover, three down-regulating proteins are directly involved in nodule process.

## Introduction

Reactive oxygen species (ROS) are biologically important O_2_ derivatives that are by-products of aerobic metabolism (Köhler et al., 2014). The most important ROS include several small chemical compounds such as the superoxide anion, hydroxyl anion (OH^–^), hydrogen peroxide (H_2_O_2_), nitric oxide (NO), and peroxynitrite (ONOO^–^) (Suvorava and Kojda, 2009). In addition, ROS function as crucial second messengers, working as redox regulators in a multitude of biological processes (Ortega-Villasante et al., 2018). Excessive production of ROS or impaired ROS detoxification causes oxidative damage to lipid, protein, and DNA (Chung et al., 2010). Oxidative damage is associated with various metabolic disorders (Luo et al., 2020). Removal of ROS by antioxidants plays an important part in limiting this damage (Oláhová et al., 2008). Intracellular antioxidants include enzymes such as superoxide dismutase, catalase, and Gpx, as well as low molecular weight antioxidants like flavonoids, glutathione (GSH) and phenolics (Liu et al., 2014). Among the detoxification enzymes, glutathione peroxidase plays an important role in scavenging reactive oxygen metabolites and protection of organisms from oxidative damage (Khanyok et al., 1997).

Glutathione peroxidase (EC 1.11.1.9) is a selenium-containing antioxidant enzyme with peroxidase activity in catalyzing the reduction of hydroperoxides by GSH (Zheng et al., 2008). Gpx catalyzes the reduction of H_2_O_2_ and organic hydroperoxides by oxidation of GSH to GSSG, which in turn is reduced by GR (Heyob et al., 2008). GSH (gamma-L-glutamyl-L-cysteinyl-glycine) is a ubiquitous low-molecular-weight, thiol-containing compound inside the cells and involved in the antioxidant defense and intracellular redox signaling (Desideri et al., 2012). GSH is present in millimolar concentrations in eukaryotic and prokaryotic cells (Fujii et al., 2011). In cells, GSH biosynthesis occurs through two ATP-dependent reactions, usually involving distinct enzymes (Albino et al., 2012). Glutamic acid are combined with cysteine by γ-glutamyl cysteine synthetase. The product γ-glutamylcysteine (γ-GC) is conjugated to glycine to form GSH by the enzyme glutathione synthetase (Cheng et al., 2017).

Rhizobia are Gram-negative nitrogen-fixing soil bacteria that can form nodules on the roots of the host family Leguminosae. During infection, rhizobia are exposed to ROS released by the oxidative burst which is an early plant defense response (Temme and Tudzynski, 2009), and the nodules are also exposed to high concentrations of ROS due to the high rate of respiration necessary to supply the energy expenditure required for the reduction of dinitrogen to ammonia by nitrogenase (Harrison et al., 2005). Therefore, rhizobia require mechanisms for scavenging excess ROS that allow them to maintain sufficient levels of cellular redox homeostasis to support symbiotic processes. GSH plays a critical role by regulating redox homeostasis, and appears to be essential for initiation and development of the root nodules (Bianucci et al., 2008; Yin et al., 2018). In *Rhizobium etli* and *Sinorhizobium meliloti*, GSH produced in the bacteroids and host cells is required for symbiotic effectiveness (Harrison et al., 2005; Taté et al., 2012; Becana et al., 2018). For *Rhizobium leguminosarum*, GSH-deficient mutant cannot efficiently utilize various compounds and induced the formation of pea nodules with poor nitrogen-fixing capacity (Cheng et al., 2017). Notably, glutathione reductase in *S. meliloti* has been recently reported to be required for both redox homeostasis and symbiosis (Tang et al., 2018). In bradyrhizobial peanut symbionts, the activity of peroxidases is counteracting an oxidative burst for the successful establishment of the symbiosis (Muñoz et al., 2016), however, to date, it has not been determined whether the Gpx-catalyzed the reduction of harmful peroxides by the oxidation of GSH to GSSG is required for intracellular redox homeostasis and symbiosis. In this study, mutation in *gpxA* gene, *gshR* gene and both *gpxA* and *gshR* genes, was constructed by homologous recombination, and the role of *R. leguminosarum* Gpx in oxidative stress and during nitrogen-fixing symbiosis was investigated by comparing the phenotypes of wild-type and mutant strains. Proteome analysis provides a more comprehensive understanding of the differences between the wild-type and *gpxA* mutant nodule bacteroids.

## Materials and Methods

### Bacterial Strains, Plasmids, and Growth Conditions

Bacterial strains, oligonucleotide primers, and plasmids used in this study are listed in Supplementary Table 1. *Escherichia coli* strains were grown in LB medium at 37°C. *R. leguminosarum* strains were grown under aerobic conditions at 28°C in acid minimal salts medium (AMS) (Poole et al., 1994) or tryptone-yeast extract (TY) medium (Beringer and Hopwood, 1976) supplemented with NH_4_Cl (10 mM) as a nitrogen source and D-glucose (10 mM) as a carbon source (AMS Glc/NH_4_^+^). The following antibiotics were added at the following concentrations (μg/mL): streptomycin (Str), 500; ampicillin (Amp), 50; spectinomycin (Spe), 100; kanamycin (Km), 20; gentamicin (Gm), 20; neomycin (Neo), 80, or 250 (for making *gshR* mutant). Optical density at 600 nm (OD_600_) was measured throughout three independent culture growth.

### Construction of the *R. leguminosarum* Mutants and Complementation

To mutate *gpxA*, primers gpxAF and gpxAR derived from the *gpxA* (*RL1698*) gene sequence were used to amplify genomic DNA specifically from *R. leguminosarum* 3841 (Johnston and Beringer, 1975), and the 2.0-kb product was inserted into pMD18-T vector, giving plasmid pMDgpxA. The spectinomycin resistance cassette from *Bam*HI and *Pst*I-digested pHP45Ωspe was first inserted into the unique *Bgl*II site of pMDgpxA, producing pMDgpxAΩSpe (Fellay et al., 1987). The *Bam*HI/*Xba*I fragment from pMDgpxAΩSpe was further inserted into pJQ200SK to create pJQgpxAΩspe (Quandt and Hynes, 1993). Plasmid pJQgpxAΩspe was conjugated into the recipient strain RL3841, and *gpxA* mutant (RLgpxA) isolated by selecting for recombination using the spectinomycin resistant and a previously described *sac* mutagenesis strategy (Kumar et al., 2005). The insertion was mapped by the PCR method using primers MgpxAF or MgpxAR together with pOTF.

To mutate *gshR*, primers gshRF and gshRR derived from the *gshR* region were used to amplify genomic DNA specifically from *R. leguminosarum* 3841. The about 600-bp *gshR* PCR product was first inserted into *Xba*I and *Hin*dIII sites of pK19mob (Schäfer et al., 1994), giving plasmid pKgshR. Using pRK2013 as helper plasmid (Figurski and Helinski, 1979), pKgshR was further conjugated with *R. leguminosarum* 3841 as previously described (Karunakaran et al., 2010). The mutations RLgpxA and RLgshR were further combined to construct double mutant as described below. To introduce the spectinomycin resistance-marked *gpxA*:Spe mutation, the phage RL38 was used to lyse wild-type strain RLgpxA (*gpxA*:Spe). Using transduction of spectinomycin resistance to RLgshR (*gshR*), strain RLgpxAgshR (*gpxA gshR*) was generated.

For complementation of the *gpxA* mutation, the wild-type *gpxA* gene fragment was amplified with cgpxAF and cgpxAR and subcloned into pBBR1MCS-5 as an *Xba*I/*Pst*I fragment (Kovach et al., 1994). Using the pRK2013 as helper plasmid, the resulting plasmid, pBBRgpxA, was conjugated into the RLgpxA recipient strain by triparental mating (Karunakaran et al., 2010), Complemented strain RLgpxA(pBBRgpxA) was isolated using selection for gentamicin resistance as previously described (Karunakaran et al., 2006).

### Assay of H_2_O_2_ Sensitivity

The sensitivity of *R. leguminosarum* to H_2_O_2_ was assessed with a filter paper disc diffusion method. The logarithmic phase (OD_600_ 0.4–0.6) *R. leguminosarum* strains were collected by centrifugation, washed twice and resuspended in sterile phosphate-buffered saline (PBS, 8.0 mM Na_2_HPO_4_, 136 mM NaCl, 1.5 mM KH_2_PO_4_, 2.6 mM KCl). 100 μL of bacterial suspensions containing 10^8^ cfu/mL was spread on agar plates made from AMS Glc/NH_4_^+^. Sterile filter paper discs (6-mm diameter) were placed on the TY media plate on which a bacterial suspension was spread, and a disc was impregnated with 30 μL of H_2_O_2_ solution. In this experiment, H_2_O_2_ was used in different concentrations (20 mmol/L, 100 mmol/L, 500 mmol/L, and 1,000 mmol/L). Water was used as a negative control. The experiment consisted of three independent experiments, and the two-way ANOVA (*P* < 0.05) was used to test for differences.

### Assay of Glutathione Levels in *R. leguminosarum*

For analysis of glutathione content, logarithmic phase cultures of mutant strains RLgpxA, RLgshR, RLgpxAgshR, and wild-type RL3841 with an optical density (OD_600_) 0.4–0.6 were collected on ice, centrifuged (5,000 rpm) for 5 min at 4°C. The cells were sonicated in an ice-water bath for 15 min. The sonicate was centrifuged (12,000 rpm, 10 min) at 4°C. GSH contents were measured spectrophotometrically as previously described (Rahman et al., 2006). The experiment was constructed from three independent experiments, and the two-way ANOVA (*P* < 0.05) was used to test for differences.

### Plant Experiments and Microscopy Studies of Nodules

Nodulation assays on pea were carried out as previously described (Cheng et al., 2017). Pea seeds were sterilized with 5% sodium hypochlorite, placed in 1 L pots containing sterile vermiculite with nitrogen-free Fahraeus solution. At the time of sowing, *R. leguminosarum* strains were inoculated with 10^7^ CFU per seed. Plants were incubated in a controlled-environment growth chamber with a 8 h at 20°C in the dark and 16 h photoperiod at 22°C in the light. For the dry weight plants were placed in 2 L beaker containing sterile vermiculite, watered with distilled nitrogen-free Fahraeus solution and harvested at 7 weeks from planting. The shoot was removed from the root and dried at 70°C in a warm incubator for 72 h (Wang et al., 2019). Nitrogenase activity of nodules at flowering (4 weeks) was assessed by acetylene reduction assay according to the method described previously (Allaway et al., 2000). The experiment was constructed from three independent experiments, and the two-way ANOVA (*P* < 0.05) was used to test for differences.

Four-week-old nodules were obtained and examined by the use of both light and electron microscopes. Nodules were fixed with cold 2.5% glutaraldehyde, and then postfixed in 1.5% osmium tetroxide after collection. Semi-thin sections (1–3 μm) of root nodules were obtaining, colored in toluidine blue, and evaluated with light microscope (SZX16, Olympus). For electron microscopy studies, ultra-thin sections were cut, double-stained with uranyl acetate and lead citrate, and photographed with a transmission electron microscope Hitachi H-7100.

### Colonization Ability in the Pea Rhizosphere

Root colonization assays were performed according to the method described previously (Cheng et al., 2017). Pea seedlings were grown in vermiculite for 1 week, and inoculated with each mutant strain and wild-type RL3841 at the cfu ratios 1,000:1,000, 0:1,000, and 1,000:0. Shoots were cut-off after 1 week (2 weeks after sowing), and the roots were added with 20 mL of sterile PBS and vortexed for 15 min (Karunakaran et al., 2006). The samples were further serially diluted 10-fold in sterile water, and the colony number was counted on TY solid medium plates containing streptomycin (for mutant and wild-type strains together) or neomycin and streptomycin (for RLgshR) or streptomycin and spectinomycin (for RLgpxA or RLgpxAgshR), giving the number of viable root – and rhizosphere-associated bacteria (Barr et al., 2008). Each treatment included 10 replications, and the two-way ANOVA (*P* < 0.05) was used to test for differences.

### RNA Extraction and Quantitative Reverse Transcription-Polymerase Chain Reaction

Quantitative reverse transcription-polymerase chain reaction (qRT-PCR) was performed to investigate the differences in the expression of various genes between mutant and wild type. *R. leguminosarum* samples were collected in triplicates from free-living cultures in AMS liquid medium, or 2, 4, 6-week-old nodules. The pea nodules were harvested, and then finely grinded into powder in liquid nitrogen. Total RNA was isolated using TRIzol reagent (Invitrogen) and measured using a Nanodrop spectrophotometer (Thermo Fisher Scientific) (Smith et al., 1985). cDNA was synthesized with the use of random hexamers and SuperScript^TM^ II reverse transcriptase. qRT-PCR assay was carried out using SYBR^®^ premix Ex Taq^TM^ (Takara, Dalian, China) on a CFX96 Real-Time System (Bio-Rad). Primer sequences for *gpxA*, *gshR*, *gyrB1*, *katG*, *katE*, *gshB*, *aapJ*, *braC* are listed in Supplementary Table 1. *Gyrb1* was used as the reference housekeeping gene. Data were obtained and analyzed according to the previously described method (Prell et al., 2009). The one-way ANOVA (*P* < 0.05) was used to test for differences.

### Protein Extraction and Trypsin Digestion

The 4-week-old nodules were dripped into liquid nitrogen and ground to a fine cell powder. The ground powder of cells was then transferred into a 5-mL ice-cold centrifuge tube, and suspended in four volumes of lysis buffer (8 M urea, 1% protease inhibitor cocktail). The slurry was sonicated for three times on ice using a high-intensity ultrasonic processor, and the lysates were cleared by centrifugation at 12,000 × *g* at 4°C for 10 min. The supernatant was collected and stored at −80°C. The total protein concentration in the supernatant was measured by using the bicinchoninic acid (BCA) protein assay kit (Pierce, Rockland, IL, United States). Proteins were first reduced by incubation with 5 mM dithiothreitol for 30 min at 56°C. For trypsin digestion, the protein solution were first alkylated with 11 mM iodoacetamide (IAM, Sigma) in the darkness at room temperature for 15 min. Then, each protein was dissolved by adding 100 mM tetraethylammonium bromide (TEAB) to reduce the urea concentration less than 2M. Finally, the proteins were digested with trypsin at 1:50 trypsin-to-protein mass ratio overnight at 37°C, and followed with trypsin at a trypsin-to-protein mass ratio of 1:100 for a second 4 h-digestion.

### Tandem Mass Tag Labeling and LC-MS/MS Analysis

After trypsin digestion was completed, the peptides were desalted using a Strata X C18 SPE column (Phenomenex). The peptides were vacuum-dried, reconstituted in 0.5 M TEAB and processed using the 6-plex tandem mass tag (TMT) kit (Thermo Fisher Scientific, Bremen, GA, United States) according to the manufacturer’s protocol. Briefly, one unit of TMT reagent (defined as the amount of reagent required to label 100 μg of protein) was thawed and extracted with 24 μL of acetonitrile. The resulting peptide mixtures were further incubated at room temperature for 2 h, pooled, desalted, centrifuged, and dried by vacuum.

The resultant tryptic peptides were crushed into various fractions by high pH reverse-phase HPLC using an Agilent 300 Extend C18 column (150 mm × 4.6 mm, 5 μm). Briefly, the peptides were first fractionated with a gradient of 2–60% (v/v) acetonitrile in 10 mM ammonium bicarbonate at pH 10 over 80 min into 80 fractions. Then, the peptides were concatenated and combined into 18 fractions, and dried using vacuum centrifugation. LC-MS/MS analysis peptides were dissolved in solvent A (0.1% formic acid in aqueous solution), directly loaded onto a reversed phase column (Acclaim PepMap 100, Thermo Fisher Scientific). Separation of peptides was performed using a reversed-phase analytical column (Thermo Fisher Scientific, Shanghai, China). The gradient was as follows: 6–22% of solution B (0.1% FA in 98% ACN) over 26 min, 23–35% in 8 min, 80% in 3 min, and finally 80% for 3 min at a constant flow rate of 400 nL/min on an EASY-nLC 1,000 ultra-performance liquid chromatography (UPLC) system (Thermo Fisher Scientific). The resulting peptides were subjected to an NSI source, and then tested by tandem mass spectrometry (MS/MS) in a Q ExactiveTM Plus (Thermo, Shanghai, China) coupled online to UPLC. Peptides were detected by the Orbitrap analyzer at a mass resolution of 70,000. The peptides were subjected to run MS/MS analysis using a setting of 28 eV normalized collision energy (NCE), and ion fragments were detected in the Orbitrap detector at a resolution of 17,500. The MS analysis alternated between MS and data-dependent MS/MS scans using 15.0 s dynamic exclusion. The applied electrospray voltage was set to 2.0 kV. Automatic gain control (AGC) was applied to prevent overfilling of the ion trap, and 5 × 10^4^ ions were accumulated for generation of the MS/MS spectra. The scan range of full MS was set from 350 to 1,800 m/z, and the fixed first mass was set to 100 m/z. The experiments were performed in triplicate.

### Data Analysis

The MS/MS data of all experiments were processed using the MaxQuant software version 1.5.2.8 (Cox and Mann, 2008). The spectra were searched against an annotated *R. leguminosarum* protein database (Young et al., 2006). Trypsin/P was treated as the cleavage enzyme and the search allowed up to four missing cleavages. The mass tolerance for precursor ions was set at 20 ppm, the final tolerance was set at 5 ppm, and the mass tolerance for precursor ions was set to 0.02 Da. Alkylation of cysteine by carbamidomethylation was set as a fixed post-translational modification, and methionine oxidation was treated as variable modifications for database search. The false discovery rate (FDR) was <1%, and a minimum Andromeda score cut-off was set at 40 for modified peptides. Protein expression data indicate statistically significant differences according to Student’s *t*-tests (*P* < 0.05). Differentially expressed proteins were defined as having at least a 1.2-fold change. The mass spectrometry proteomics data and the Supplementary Data have been uploaded to the ProteomeXchange Consortium via the PRIDE partner repository with the dataset identifier PXD022029.

### Bioinformatics Analysis

Sequence similarity searches were performed with FASTA against current releases of the Uniprot Knowledgebase (UniProtKB). The gene ontology (GO) categories for subcellular location were derived from UniProt and *Rhizobium* database entries. The metabolic and biological processes were studied by using Kyoto Encyclopedia of Genes and Genomes (KEGG) database.

## Results

### Growth of *R. leguminosarum* Mutants Under Free-Living Conditions

The whole genome sequence analysis of wild-type RL3841 revealed the presence of a glutathione peroxidase gene (*gpxA*) and a glutathione reductase encoding gene (*gshR*). *gpxA* (*RL_RS08810*, *RL1698*) is predicted to encode a polypeptide of 182 amino acids with a molecular mass of about 20.02 kDa and a theoretical pI value of 5.51, and *gshR* (*RL_RS13915*, *RL2694*) is predicted to encode a polypeptide of 461 amino acids with a molecular mass of about 50.05 kDa and a theoretical pI value of 5.87. To experimentally confirm the potential function of these two redox genes, single and double mutants were made by means of mutagenesis. The mutants were first evaluated for growth in liquid AMS minimal medium. None of the mutant strains presented a substantial reduced growth rate (Supplementary Figure 1). These results suggest neither *gpxA* gene nor *gshR* gene has effect on *R. leguminosarum* growth in AMS minimal medium, and contrast with the data from *S. meliloti* in which glutathione reductase is important for the growth (Tang et al., 2018).

### Antioxidation Analysis of *R. leguminosarum* Mutants

In order to study the sensitivity to oxidative stress, growth of *R. leguminosarum* mutants and wild-type strain was monitored by carrying out disk diffusion assays (Table 1). The growth of mutants RLgpxA and RLgshR was not significantly affected by 20 mmol/L H_2_O_2_ treatment, as compared with the wt RL3841 strain. However, at concentrations of 100, 500, and 1,000 mmol/L, the RLpxA and RLgshR mutant strains presented a clear sensitivity to the H_2_O_2_ treatment, and their growth was significantly inhibited. The results suggest that the GpxA and GshR could play an important role in protecting cells from hydrogen peroxide stress. Moreover, at H_2_O_2_ concentration of 20, 100, and 500 mmol/L, the double mutant RLgpxAgshR, in which both Gpx and GR are inactivated, presented a slightly enhanced sensitivity, but significantly inhibited growth rate compared to the single mutant strains, suggesting that double mutation in Gpx and GR increased the sensitivity of *R. leguminosarum* strains to H_2_O_2_.

**TABLE 1:** The inhibition diameters of *R. leguminosarum* strains to different concentrations of H_2_O_2_.

**Concentration of H_2_O_2_ (mmol/L)**	**Diameter (cm)**				
	**3841**	**RLgshR**	**RLgpxA**	**RLgpxAgshR**	**RLgpxA (pBBRgpxA)**
20	3.40 ± 0.40^*a*^	4.07 ± 0.21^*a*^	4.03 ± 0.12^*a*^	4.93 ± 0.21^*b*^	3.58 ± 0.61^*a*^
100	6.53 ± 0.35^*a*^	10.87 ± 0.38^*b*^	9.87 ± 0.35^*b*^	12.23 ± 0.31^*c*^	6.90 ± 0.52^*a*^
500	13.57 ± 0.35^*a*^	17.60 ± 0.46^*b*^	16.50 ± 0.26^*b*^	19.13 ± 0.81^*c*^	14.22 ± 0.78^*a*^
1,000	18.63 ± 0.40^*a*^	21.53 ± 0.42^*b*^	20.90 ± 0.10^*b*^	22.83 ± 0.15^*b*^	19.15 ± 0.64^*a*^

*Data (mean ± SEM) are the averages of three independent experiments. ^*a,b,c*^Values with different letters are significantly different from that of the wild-type RL3841 control (two-way ANOVA, *P* < 0.05).*

### Effect of Mutant on the Concentration of GSH

**FIGURE 1:**
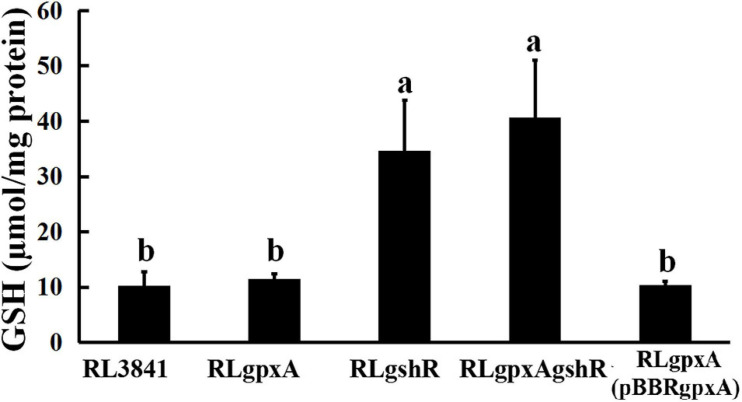
The accumulation of reduced glutathione in *R. leguminosarum* strains. Data are from three biological samples (±SEM). ^*a,b*^Different letters indicates the value is significantly different (two-way ANOVA, *P* < 0.05).

The GSH contents were quantified spectrophotometrically. The data showed that the content of GSH has no significant effect in the *gpxA* mutant, compared to wild-type strain, while it was 2.5-fold higher in both *gshR* mutant and double mutant (Figure 1). These results indicate that a mutant in *gshR* gene increased GSH levels.

### Transcriptional Analysis of the Relative Gene Expression

Both Gpx and GR play an important role in anti-oxidation responses, and catalase activity can function as a marker to assess whether rhizobia are under oxidative stress (Tang et al., 2018), which prompted us to study the expression of the catalase genes by qRT-PCR. Expression of *katG* and *katE* in RLgpxA were increased 6.77- and 4.07-fold compared with levels in RL3841, while their expression in RLgshR were decreased 4.35- and 14.29-fold (Table 2). However, in the double mutant, both *katG* and *katE* expression showed no significance, suggesting a complex interrelation of the glutathione peroxidase/reductase (GSH-Px/GSSG-Rd) enzyme system. The level of reduced GSH was no significant influence in the *gpxA* mutant, bur strikingly increased in the *gshR* mutant, qRT-PCR was performed to measure the expression level of glutathione synthetase gene *gshB*. The data showed that the expression of *gshB* was increased in the *gshR* mutant and the double mutant compared with the parent strain but there was no statistically significant difference in the *gpxA* mutant (Table 2).

**TABLE 2:** Expression of genes in *R. leguminosarum* strains growing in AMS medium by qRT-PCR analysis.

**Strain**	**Gene expression** ^α^				
	***katG***	***katE***	***gshB***	***aapJ***	***braC***
RLgpxA	6.77 ± 0.51*	4.07 ± 2.43*	1.21 ± 0.73	0.04 ± 0.05*	0.04 ± 0.06*
RLgshR	0.23 ± 0.12*	0.07 ± 0.01*	0.17 ± 0.05*	0.64 ± 0.43	0.47 ± 0.16
RLgpxAgshR	1.51 ± 0.44	0.59 ± 0.35	0.37 ± 0.13*	0.30 ± 0.07*	0.37 ± 0.14*

^α^The expression of these genes in mutant strains were compared with RL3841. Bacteria were grown in AMS Glc/NH_4_^+^ overnight. Asterisk (*) indicates gene expression was at least two-fold different from RL3841. Values are given mean ± standard error of the mean (±SEM).

GSH was reported to be involved in the activity of amino acid transporters *R. leguminosarum* (Cheng et al., 2017). To examine its possible mechanism, expression of the two ABC transporters, the general amino acid permease (Aap) and the branched-chain amino acid permease (Bra), was detected by qRT-PCR. Expression of *aapJ* and *braC* in RLgpxA were both decreased 25-fold compared with levels in RL3841, while expression of these two genes in RLgshR did not alter significantly (Table 2). Moreover, as there was slightly but significantly decreased in expression of either *aapJ* or *braC* in double mutant RLgpxAgshR, it suggests that the transporter defect caused by the GpxA-deficiency was partly restored by mutant in the *gshR* gene.

Expression of the *gpxA* and *gsh*R genes in root nodules collected at 14, 28, and 42 days post inoculation was also detected by qRT-PCR, but no differences were found comparison to that in free-living cells (Supplementary Table 2).

### Rhizosphere Colonization and Competition by *R. leguminosarum* Strains

**FIGURE 2:**
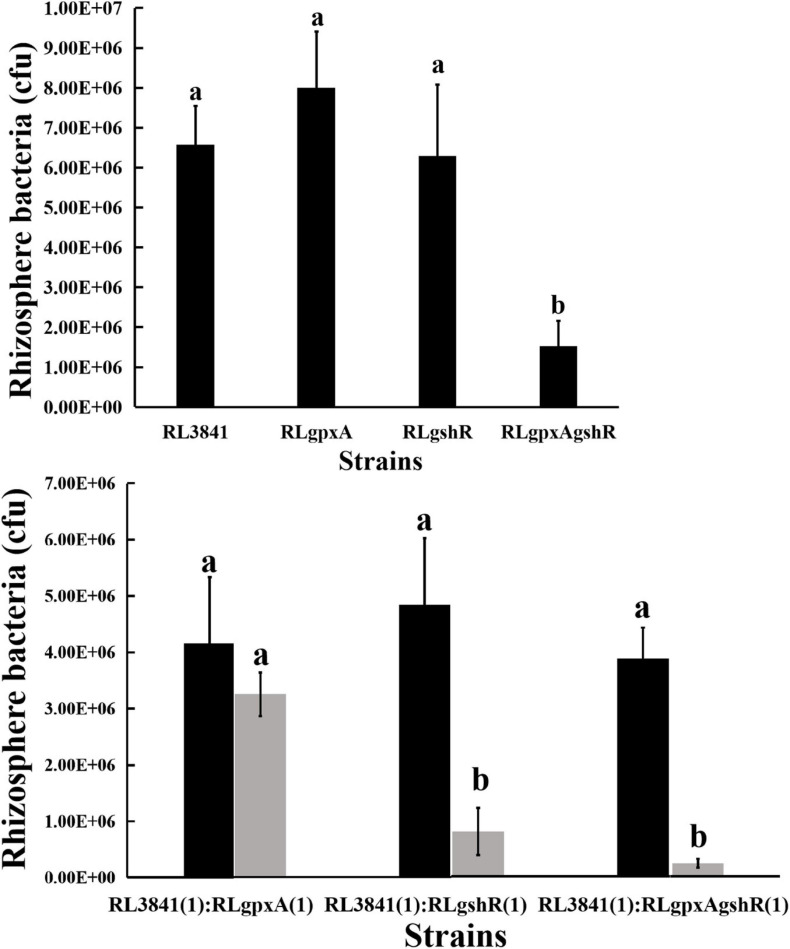
Competition of the wild-type RL3841 (black bars) and the mutants (gray bars) in the pea rhizospheres. The *x*-axis indicates the initial inoculation ratios, with 1 corresponding to 1 × 10^3^ CFU. The number of colony-forming bacteria (per plant) from 10 plants are shown. ^*a,b*^Values with different letters are significantly different between mutant and wild-type control (two-way ANOVA, *P* < 0.05).

It has been reported that the *R. leguminosarum gshB* mutant is severely impaired in rhizosphere colonization (Cheng et al., 2017). Therefore, the colonization ability of *R. leguminosarum* mutants for growth in the rhizosphere of the plant was evaluated by inoculating soils of a low microbial population (10^3^ or 10^4^ bacteria per seedling) into the short-term colonization of the plant rhizosphere, and measuring the total count of bacteria after 1 week. When the single mutants RLgpxA, RLgshR, and the wild-type RL3841 were inoculated alone into a pea rhizosphere, there was no significant difference (*P* < 0.05) in the numbers of bacteria (Figure 2). However, the ratio between double mutant RLgpxAgshR and wild-type 3841 was 23.14%, and was at a significant disadvantage compared to the single mutants RLgpxA, RLgshR, and the wild-type RL3841. When inoculated together in equal proportion, mutation of *gpxA* did not affect *R. leguminosarum* rhizosphere competitive capacities. In contrast, the *gshR* mutant RLgshR and the double mutant RLgpxAgshR were at a significant disadvantage (16.57 and 6.10% of bacteria recovered) compared to the wild-type RL3841 (Figure 2). The results showed that GshR was essential for competition in the host plant rhizosphere by *R. leguminosarum*, while GpxA had an enhanced effect on the defect of the rhizosphere colonization and competition by the *gpxA* and *gshR* double mutant.

### Symbiotic Phenotypes of *R. leguminosarum* Strains

**FIGURE 3:**
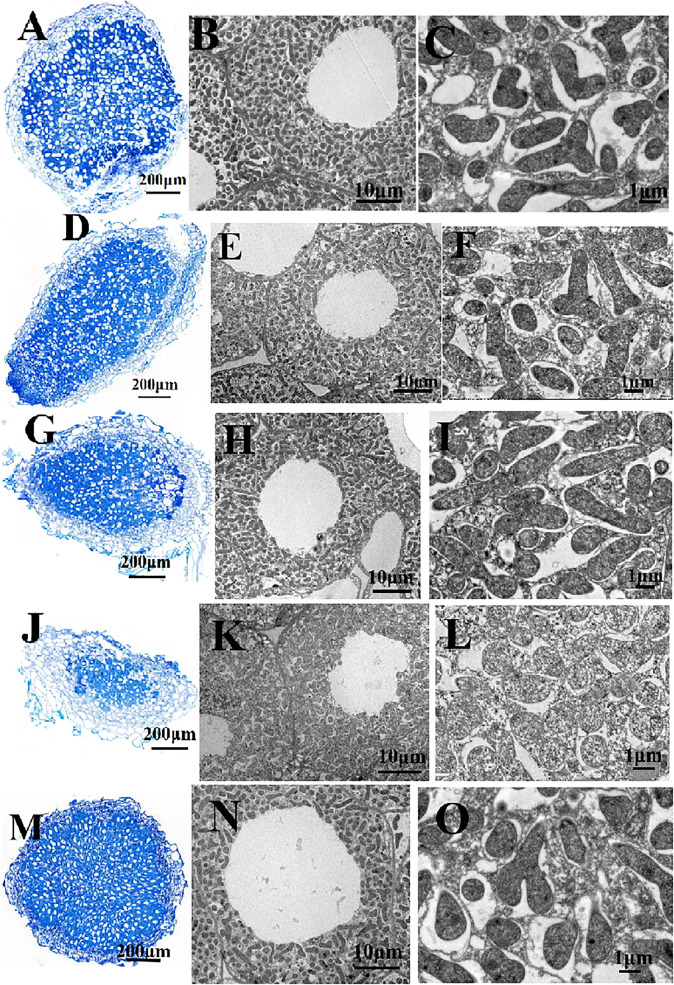
Structure of pea nodules and bacteroids. Root nodules were induced by RL3841 **(A–C)**, RLgshR **(D–F)**, RLgpxA **(G–I)**, RLgshRgpxA **(J–L)**, RLgpxA(pBBRgpxA) **(M–O)**. Scale bars = 200 μm **(A,D,G,J,M)**, 10 μm **(B,E,H,K,N)**, 1 μm **(C,F,I,L,O)**.

Pea seedlings were inoculated with *R. leguminosarum* mutants and wild-type RL3841, and after 4 weeks, acetylene reduction activity and nodule numbers per plant were measured. RLgshR formed nodules with normal size. However, RLgpxA and double mutant RLgpxAgshR resulted in smaller nodule structures and reduction in nodule fresh weight (Table 3). There was no statistically significant difference in the number of nodules per plant between plants inoculated mutants RLgpxA, RLgshR, RLgpxAgshR and plants inoculated with wild-type RL3841 (Table 3). Mutation in *gshR* gene induced nodules with the same capacity for acetylene reduction as nodules infected by wild-type RL3841. In contrast, plants inoculated with RLgpxA and double mutant RLgpxAgshR showed a significantly more than 50% decrease in acetylene reduction activity compared to the wt RL3841, and no significant difference was found between these two mutant strains (Table 3). At 7 weeks post inoculation, the dry weight values of plants inoculated with RLgpxA and double mutant RLgpxAgshR was no more than 56% of that of plants inoculated with wild-type RL3841 (Table 3). When the recombinant plasmid pBBRgpxA was introduced into mutant RLgpxA, plants inoculated with the complemented strain RLgpxA(pBBRgpxA) elicited normal nodules and had the ability to fix nitrogen at the same rate as wt RL3841 inoculated plants (Figures 3M–O and Table 3).

**TABLE 3:** Symbiotic behavior of *R. leguminosarum* mutants.

**Strain**	**Nodules per plant**	**Fresh nodule weight per plant (mg)**	**Acetylene reduction (μmoles acetylene per plant per h)**	**Dry weight per plant (g)**
RL3841	110.7 ± 11.1^*a*^	76.89 ± 7.75^*a*^	2.07 ± 0.37^*a*^	1.64 ± 0.13^*a*^
RLgpxA	102.7 ± 13.0^*a*^	42.27 ± 6.62^*b*^	0.97 ± 0.20^*b*^	0.91 ± 0.14^*b*^
RLgshR	108.8 ± 11.6^*a*^	71.76 ± 6.35^*a*^	1.75 ± 0.46^*a*^	1.63 ± 0.17^*a*^
RLgpxAgshR	100.1 ± 9.3^*a*^	35.60 ± 8.33^*b*^	0.73 ± 0.09^*b*^	0.87 ± 0.13^*b*^
RLgpxA(pBBRgpxA)	108.1 ± 10.7^*a*^	68.53 ± 4.10^*a*^	1.82 ± 0.18^*a*^	1.51 ± 0.11^*a*^

*Data (mean ± SEM) are the averages of ten independent plants. ^*a,b*^Values with different letters are significantly different from that of wild-type control (two-way ANOVA, *P* < 0.05).*

Nodules were collected and examined by combined light and transmission electron microscopy. The wt RL3841 and mutant RLgshR contained normal spherical shape of the nodules (Figures 3A,D). However, microscopic analysis of the nodules obtained with RLgpxA and double mutant RLgpxAgshR showed that they contained an abnormally thick cortex (Figures 3G,J), suggesting GpxA had an important role in the formation of normal nodules. **Ultrastructural** changes **of the infected cells** and the bacteria in pea root **nodules** were observed by **transmission electron microscopy**. **Nodule cells infected by mutant** RLgshR and wt RL3841 **were occupied by** large T-shaped, Y-shaped, and rod-Shaped differentiated bacteroids (Figures 3B,C,E,F), while **nodule cells infected by mutants** RLgpxA and RLgpxAgshR consisted of a **small amount** of rod-Shaped bacteroids, a **large amount** of undifferentiated spherical-shaped bacteroids (Figures 3H,I,K,L), suggesting a general lack of differentiation into mature bacteroids.

### Proteomic Analysis of Differential Protein Expression

Proteomics analysis identified a total of 2,457 peptides with molecular weights ranging from 7 to 320 kDa. A total of twenty-seven proteins (*P* < 0.05) were identified that were differentially expressed between the mutant RlgpxA bacteroids and wild-type RL3841 bacteroids (Table 4). Among them, 20 proteins were down-regulated in *gpxA* mutant nodule bacteroids and seven proteins were up-regulated (Table 4). Subcellular localization of the 27 differentially expressed proteins showed that fourteen proteins were localized at the cytoplasm, nine proteins were localized in the periplasmic space, two proteins were located in the outer membrane, one protein was existed in the inner membrane, and one was extracellular protein (Table 4). Eight (29.63%) differential genes were localized on the megaplasmids pRL8 (*n* = 1, 3.70%), pRL9 (*n* = 2, 7.41%), pRL10 (*n* = 3, 11.11%), and pRL12 (*n* = 2, 7.41%). No differential genes were localized on the megaplasmids pRL11 and pRL7.

**TABLE 4:** Differential expression proteins in mutant-induced nodule bacteroids relative to wildtype-induced nodule bacteroids.

**Gene ID**	**Gene name**	**Subcellular localization**	**Protein description**	**MW [kDa]**	**Ratio**	***P* value**
*RL2366*	*ftsX*	Inner membrane	FtsX-like permease family protein	42.49	1.26	0.0226
*pRL100146*		Periplasmic	Transcriptional regulator	23.94	1.24	0.0238
*RL2699*	*gph*	Cytoplasmic	Phosphoglycolate phosphatase	25.93	1.22	0.0447
*RL0810*		Cytoplasmic	Polysaccharide synthesis	14.45	1.21	0.0201
*pRL100106*		Periplasmic	Uncharacterized protein	16.72	1.29	0.0036
*RL2820*		Cytoplasmic	Uncharacterized protein	8.97	1.26	0.0121
*RL1124*		Cytoplasmic	Uncharacterized protein	12.33	1.22	0.0136
*RL4655*	*intA*	Periplasmic	Solute binding protein of inositol ABC transporter	32.96	0.82	0.0005
*pRL120351*		Periplasmic	Substrate binding component of ABC transporter	49.96	0.82	0.0001
*pRL120071*		Periplasmic	Substrate-binding component of ABC transporter	30.48	0.78	0.0282
*RL4417*		Periplasmic	Solute-binding component of ABC transporter	70.67	0.76	0.0061
*RL1499*	*ropA2*	Outer membrane	Porin	36.71	0.74	0.0008
*RL2775*	*ropA1*	OuterMembrane	Porin	36.80	0.72	0.0076
*RL3066*		Periplasmic	Transmembrane protein	17.22	0.69	0.0083
*RL2554*		Periplasmic	Exported protein	12.42	0.65	0.0291
*RL4392*	*fdsB*	Cytoplasmic	NAD-dependent formate dehydrogenase beta subunit	54.75	0.82	0.0485
*pRL90175*	*bdhA*	Cytoplasmic	D-beta-hydroxybutyrate dehydrogenase like protein	27.21	0.82	0.0473
*RL0644*	*rbtD*	Cytoplasmic	Ribitol 2-dehydrogenase	25.73	0.8	0.0419
*RL2323*		Periplasmic	The glucose-fructose oxidoreductase/inositol dehydrogenase/rhizopine catabolism protein (GFO/IDH/MocA) dehydrogenase	38.70	0.76	0.0091
*RL0802*		Cytoplasmic	Deoxygenase	31.23	0.8	0.0469
*RL0866*	*glcF*	Cytoplasmic	Glycolate oxidase iron-sulfur subunit	46.87	0.79	0.0089
*pRL80022*		Cytoplasmic	Alpha/beta hydrolase	35.12	0.78	0.0461
*RL3549*	*glnII*	Cytoplasmic	Glutamine synthetase	38.76	0.64	0.0118
*RL1559*	*acpP*	Cytoplasmic	Acyl carrier protein	8.37	0.83	0.0301
*pRL100169*	*rhiA*	Extracellular	Rhizosphere expressed protein RhiA	24.89	0.82	0.0018
*RL0843*	*rlmE*	Cytoplasmic	Ribosomal RNA large subunit methyltransferase E	26.45	0.81	0.0306
*pRL90221*		Cytoplasmic	Uncharacterized protein	28.31	0.71	0.0310

Among the seven up-regulated genes in nodules induced by the *gpxA* mutant, *RL2366* codes for FtsX, which is an ABC transporter essential for efficient cell division in *E. coli*; *pRL100146* codes for a transcriptional regulator; *RL2699* and *RL0810* code for a phosphoglycolate phosphatase and a polysaccharide synthesis, respectively (Table 4). In contrast, among the twenty down-regulated genes in nodules induced by the *gpxA* mutant, eight genes *RL4665*, *pRL120351*, *pRL120071*, *RL4417*, *RL1499*, *RL2775*, *RL3066*, and *RL2554* code for membrane transport proteins, of which four were ABC-type transporters. Four genes *RL4392*, *pRL90175*, *RL0644*, and *RL2323* code for dehydrogenases. *RL0802*, *RL0866*, and *pRL80022* code for a deoxygenase, an oxidase and a hydrolase, respectively. Many of the differentially expressed genes display similarity to genes known to be involved in cellular metabolism and membrane transport processes. The number of differentially expressed metabolism-associated proteins indicated that Gpx played important roles in a wide variety of important metabolic processes by regulating the expression of genes encoding transporters, key enzymes, and other proteins involved in metabolic homeostasis. In addition, *RL3549* codes for glutamine synthetase involved in assimilation of ammonium, *RL1559* codes for the acyl carrier protein required for the biosynthesis of lipid A, and *pRL100169* is a rhizosphere-expressed gene *rhiA*. These three genes have been reported by others to be functionally linked with symbiosis (Table 4) (Kannenberg and Carlson, 2001; Yadav et al., 2002; Crespo-Rivas et al., 2019). Finally, *RL0843* codes Ribosomal RNA large subunit methyltransferase E, which is involved in ribosomal RNA maturation, and the loss of GpxA resulted in the differential expression of proteins (*pRL100106*, *RL2820*, *RL1124*, and *pRL90221*) of unknown function.

## Discussion

The presence of GSH is likely to mean that requirement for the redox enzyme within the cell is crucially important to *Rhizobium* (Muglia et al., 2008; Taté et al., 2012). In *R. leguminosarum*, GSH plays a central role in both the growth of free-living bacteria and symbiotic nitrogen fixation (Cheng et al., 2017). Regulation of intracellular GSH/GSSG is a function of the flux through the two enzymes of the GSH redox cycle, Gpx and GR. In this study, the enzymes Gpx and GR were studied both alone and in combination. Our findings suggest as the key enzymes in the redox metabolism of GSH, both Gpx and GR are important for the cellular redox. However, the results also suggest that Gpx, but not GshR, is essential for efficient nodulation.

Glutathione has been reported to be required for the carbon uptake and has an absolute requirement for GSH for growth of *R. leguminosarum* 3841 (Cheng et al., 2017). Therefore, we hypothesized that Gpx or GR would have an additional function in cell growth. However, the growth rates and lag phases of *R. leguminosarum* 3841 were not affected by mutation in *gpxA* or *gshR* genes (Supplementary Figure 1). Although previous experimental evidences for reciprocal relationships between bacterial growth rate and Gpx ability remain unclear, mutation in glutathione reductase was able to influence markedly the growth of *S. meliloti* and *Lactobacillus sanfranciscensis* (Jänsch et al., 2007; Tang et al., 2018). According to the results of this study, we can suggest that besides through the Gpx/GR pathway, there must be another mechanism by which GSH regulates transport. In fact, it may well happen because a *gor* (GSH reductase) mutant of *R. etli* exhibited normal growth rate in medium with glucose and different amino acids (except glutamine) as sole organic source of carbon and energy (Taté et al., 2012).

Glutathione might activate a glutathione-glutathione reductase-glutathione peroxidase system to catalyze the reduction of H_2_O_2_ (Li et al., 2003). The effect of H_2_O_2_ stress under different concentration were investigated in this paper. As expected, mutants defective in both *gpxA* gene and *gshR* gene caused an increased sensitivity to H_2_O_2_ compared to wt strain RL3841. Insertion mutants of glutathione reductase gene were reported to be involved in defense against different oxides in *R. etli* and *S. meliloti*, with the former involved in H_2_O_2_ detoxification and the latter involved in diamide detoxification (Tatè et al., 2004; Tang et al., 2018). However, to date there have been no reports on the possible H_2_O_2_ detoxification for Gpx in rhizobia. Gpx is required for *R. leguminosarum* adaptation to oxidative stress, which could be due to the reaction of hydrogen peroxide with reduced GSH to form GSSG. To further study the underlying mechanism, intracellular levels of GSH and expression of two catalase genes (*katG* encoding for bifunctional catalase-peroxidase and *katE* encoding for monofunctional catalase) were analyzed. However, mutants in *gpxA* and *gshR* genes showed the opposite results. Mutant in GpxA had no significant effect on the content of GSH, but can increase the expression of the catalase genes, while mutant in GshR increased the content of GSH, which conversely suppresses expression of the catalase genes (Figure 1 and Table 2). Thus, there is likely to be complex influence of Gpx or GR on *R. leguminosarum* antioxidation, and it is not possible to conclude whether Gpx or GR is more important for antioxidation.

The role of the glutathione peroxidase/reductase enzyme system in nitrogen-fixing was investigated in nodulation of pea (*Pisum sativum*) after inoculation with *R. leguminosarum* strains. There was no statistically significant difference (*P* > 0.05) in numbers of pea nodules infected with the mutants RLgpxA, RLgshR, and RLgpxAgshR versus the parent strain at 21 dpi. Similarly, numbers of nodules on plants inoculated with *S. meliloti gor* and *R. leguminosarum gshB* mutants were similar to that inoculated with the wild-type strains (Cheng et al., 2017; Tang et al., 2018). The Gpx-deficient mutant elicited root nodules with a large decrease in the nitrogen-fixing activity (reduced to 53%). This defect in nodulation was characterized by the formation of the undeveloped nodules on pea, which is similar to the nodules induced by *R. tropici gshB* mutant on common bean (Muglia et al., 2008). Although the *R. leguminosarum gshR* mutant RLgshR cannot compete efficiently in the rhizosphere environment with wild-type RL3841, it formed normal nitrogen-fixing nodules on pea. The fact that mutation in *gor* gene of *S. meliloti* and *gshB* gene of *R. leguminosarum* was sufficient to affect bacterial growth and efficient nodulation, it might be hypothesized that the slow-growth defect of mutant strains can alter bacterial infection and plant nodulation (Cheng et al., 2017; Tang et al., 2018). Nevertheless, neither *gpxA* single mutant nor *gpxA* and *gshR* double mutant have differing effects on growth of *R. leguminosarum* 3841. Moreover, the *R. leguminosarum gshB* mutant was reported to be severely impaired in rhizosphere colonization, in contract, its *gpxA* mutant was normally grown in the rhizosphere environment. Furthermore, *gpxA* mutants of *R. leguminosarum* elicited the formation of nodules with lack of differentiation into mature bacteroids, while *R. leguminosarum gshR* formed normal-size nodules. Therefore, from the role of two glutathione enzymes investigated in the context of the differences in symbiotic phenotype, we may conclude that they might function in the different symbiotic stages, GpxA is involved in nitrogen-fixing bacteroid differentiation during pea nodule development, while GR is responsible for rhizosphere colonization at the early stage of symbiosis.

Previous reports indicated that elimination of hydrogen peroxide by glutathione peroxidase is a process of post-translational modification (Rhee et al., 2005), and many of these modifications are known to play central roles in the regulation of protein function. Henceforth, we performed a quantitative proteomic analysis to compare the protein profiles between the root bacteroids infected by *gpxA* mutant and wt RL3841. First, lack of GSH has been reported to lowers the transport rates of ABC transport systems (Tatè et al., 2004), similar result also happened in the *gpxA* mutant. Lack of GpxA reduced transcription of the Aap and Bra transporters under normal growth and down regulated eight transport proteins by proteomic analysis of nodule bacteroids (Tables 2, 4). These suggest that Gpx plays an important role in legume-rhizobial symbiosis and nodule development by maintaining the level of transport activity. Secondly, mutation in Gpx enzyme decreased the activity of redox enzymes such as dehydrogenases, deoxygenases, oxidase and hydrolase. Those enzymes were able to effectively reduced intracellular ROS production and involved in redox balance and respiration. Thirdly, three down-regulation proteins are directly involved in nodule process. Among them, glutamine synthetase has been reported to be involved in the nitric oxide signaling responses and play an important role in root nodules of *Medicago truncatula* by contributing to the antioxidant defenses system (Silva and Carvalho, 2013). Rhizobial acyl carrier protein (AcpP) was required for the biosynthesis of the flavonoid-inducible nodulation protein NodF (Geiger and López-Lara, 2002). In *R. leguminosarum* and *S. meliloti*, RirA participates in iron-dependent regulation, its mutant is hypersensitivity to oxidants and induced nodules accompanied by a significant reduction in the number of bacteria (Crespo-Rivas et al., 2019). Finally, *RL0843* codes a ribosomal methyltransferase E, which contributes to RNA processing and modification. No studies have been reported that it was involved in rhizobial symbiosis or antioxidant function, but in thermophilic bacteria, this modification is important for environmental adaptation (Kuratani et al., 2014). Overall, these data reveal *R. leguminosarum* Gpx is required for redox homeostasis and symbiosis by involving the activity of transport systems, antioxidant and nodule function.

## Data Availability Statement

The datasets presented in this study can be found in online repositories. The names of the repository/repositories and accession number(s) can be found below: https://www.ebi.ac.uk/pride/archive/projects/PXD022029.

## Author Contributions

GC conceived the idea and designed the experiments. AH, XC, JX, and SL performed the experiments. GC, QZ, DH, and XL analyzed the data. GC, AH, and XC wrote the manuscript. All authors have read and approved the final version of the manuscript.

## Conflict of Interest

The authors declare that the research was conducted in the absence of any commercial or financial relationships that could be construed as a potential conflict of interest.
