# Corrigendum: *Rhizobium leguminosarum* Glutathione Peroxidase Is Essential for Oxidative Stress Resistance and Efficient Nodulation

**DOI:** 10.3389/fmicb.2021.789870

**Published:** 2021-12-15

**Authors:** Aiqi Hu, Xiaohong Chen, Sha Luo, Qian Zou, Jing Xie, Donglan He, Xiaohua Li, Guojun Cheng

**Affiliations:** Hubei Provincial Engineering and Technology Research Center for Resources and Utilization of Microbiology, College of Life Sciences, South-Central University for Nationalities, Wuhan, China

**Keywords:** *Rhizobium leguminosarum*, glutathione peroxidase, antioxidant function, symbiotic nitrogen fixation, quantitative proteomics

In the original article, there was a mistake in ^**^Figure 3. Structure of pea nodules and bacteroid^**^ as published. ^**^Figure 3M was wrong^**^. The corrected ^**^Figure 3. Structure of pea nodules and bacteroid^**^ appears below.

**Figure 3:**
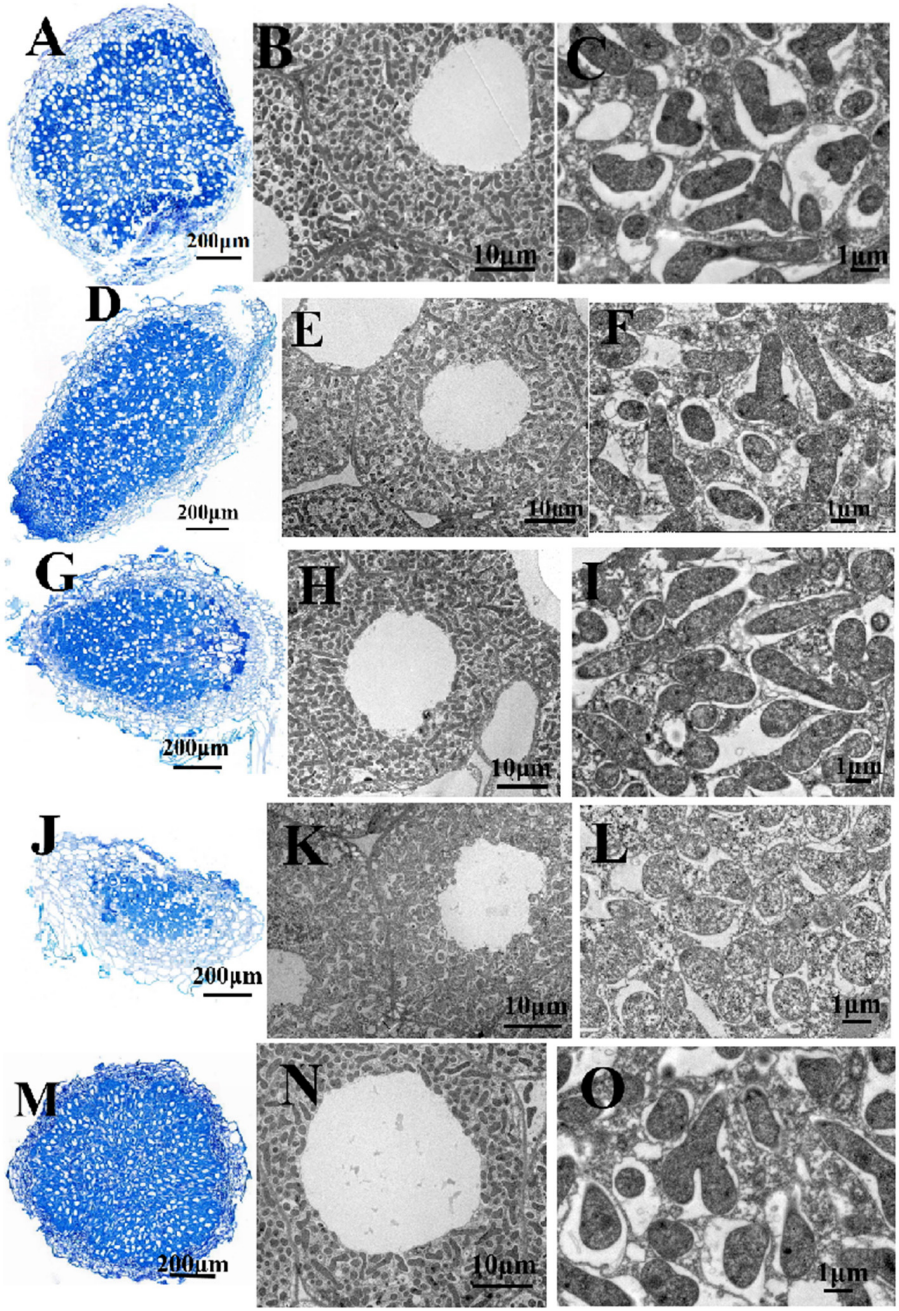
Structure of pea nodules and bacteroids. Root nodules were induced by RL3841 **(A–C)**, RLgshR **(D–F)**, RLgpxA **(G–I)**, RLgshRgpxA **(J–L)**, RLgpxA(pBBRgpxA) **(M–O)**. Scale bars = 200 μm **(A,D,G,J,M)**, 10 μm **(B,E,H,K,N)**, 1 μm **(C,F,I,L,O)**.

The authors apologize for this error and state that this does not change the scientific conclusions of the article in any way. The original article has been updated.

## Publisher's Note

All claims expressed in this article are solely those of the authors and do not necessarily represent those of their affiliated organizations, or those of the publisher, the editors and the reviewers. Any product that may be evaluated in this article, or claim that may be made by its manufacturer, is not guaranteed or endorsed by the publisher.

