# Supplementary material for: Rhizobium leguminosarum Glutathione Peroxidase Is Essential for Oxidative Stress Resistance and Efficient Nodulation
Source: Front Microbiol. 2021 Feb 9;12:627562. doi: 10.3389/fmicb.2021.627562 (PMC7900000; doi:10.3389/fmicb.2021.627562)
Supplement: Supplementary file 1 [file Data_Sheet_1.docx]

**Supplementary Table S1 Strains, plasmids and primers.**

| **Strains** | **Description** | **Reference, Source, Sequence** |
| --- | --- | --- |
| RL3841 | *Rhizobium leguminosarum* bv *viciae*; Str^r^ | Johnston and Beringer, 1975 |
| RLgpxA | RL3841 *gpxA*::ΩSpe; Str^r^ Spe^r^ | This study |
| RLgshR | RL3841 *gpxA*::pK19mob; Str^r^ Neo^r^ | This study |
| RLgpxAgshR | RL3841 *gpxA*::ΩSpe,*gpxA*::pK19mob; Str^r^ Spe^r^ Neo^r^ | This study |
| RLgpxA (pBBRgpxA) | RLgpxA carrying *gpxA* gene; Str^r^ Spe^r^ Gm^r^ | This study |
| **Plasmids** |  |  |
| pMD18-T | Vector for cloning PCR products; Amp^r^ | Takara Company |
| pJQ200SK | pACYC-derivative, P15A origin of replication, Gm^r^ | Quandt and Hynes, 1993 |
| pHP45ΩSpe | pBR322-derivative carrying ΩSpe, pHP45 replicon, Spe^r^ | Fellay et al., 1987 |
| pRK2013 | Helper plasmid for mobilizing plasmids; Km^r^ | Figurski and Helinski, 1979 |
| pBBR1MCS-5 | Broad-host-range cloning plasmid, Gm^r^ | Kovach et al., 1994 |
| pK19mob | pK19 derivative *lacZ mob*; Km^r^ | Schäfer et al., 1994 |
| pMDgpxA | gpxAF /gpxAR PCR product of *gpxA* cloned in pMD18T; Amp^r^ | This study |
| pMDgpxAΩSpe | *ΩSpec* cassette cloned into *Bgl*II site in pMDgpxA; Amp^r^ Spec^r^ | This study |
| pJQgpxAΩspe | *Xba*I/BamHI fragment from pMDgpxAΩSpe cloned in pJQ200SK; Gm^r^ Spe^r^ | This study |
| pKgshR | gshRF/gshRR PCR product of *gshR* cloned in pK19mob; Km^r^ | This study |
| pBBRgpxA | cgpxAF/cgpxAR PCR product in pBBR1MCS-5; Gm^r^ | This study |
| **Primer*** |  |  |
| gpxAF | Sense primer for *gpxA* mutation | AAATCTAGACGGAGCGCCTGCCTTTCTTCA |
| gpxAR | Antisense prime for *gpxA* mutation | AAAGGATCCGCCCGCCGCCTGAAAGCCAGC |
| MgpxAF | Sense mapping PCR primer for *gpxA* gene | GATCCGGTAGAGCAATTCGC |
| MgpxAR | Antisense mapping PCR primer for *gpxA* gene | TCACGCCGGCAGGTTCAGAT |
| gshRF | Sense mapping PCR primer for *gshR* gene | AAATCTAGATGCGTGCCGAAAAAGCTCTT |
| gshRR | Antisense mapping PCR primer for *gshR* gene | AAAAAGCTTAGCGTGTCATGGCAGAGGAT |
| MgshR | Mapping PCR primer for gshR gene | TTCGGTGTTCGGATCGCGCC |
| pOTF | Mapping primer for Ω insertion | CGGTTTACAAGCATAAAGC |
| pK19A | pK19mob mapping primer | ATCAGATCTTGATCCCCTGC |
| pK19B | pK19mob mapping primer | GCACGAGGGAGCTTCCAGGG |
| cgpxAF | Sense primer for *gpxA* complementation | TTTCTGCAGAGCTGAGGAGGTGATGTTGG |
| cgpxAR | Antisense prime for *gpxA* complementation | TTTTCTAGATTTTTTCCTATCGACGCCTC |
| QgpxAF | Sense primer for qRT-PCR of *gpxA* | AAACAGTGGATGGTCGTGAG |
| QgpxAR | Antisense primer for qRT-PCR of *gpxA* | GAGCTTTTCCAATCCCTCAT |
| QgshRF | Sense primer for qRT-PCR of *gshR* | CGCCGCCTCGCTCGGCAAGA |
| QgshRR | Antisense primer for qRT-PCR of *gshR* | ATGCTCATGGAACTGCGAAG |
| gyrB1-F | Sense primer for qRT-PCR of *GyrB1* | GGCATCACCAAAAGGGAAAA |
| gyrB1-R | Antisense primer for qRT-PCR of *GyrB1* | GCGAGGAGAATTTCGGATCA |
| QkatG-F | Sense primer for qRT-PCR of *katG* | GCAACTATTACGTCGGTCTG |
| QkatG-R | Antisense primer for qRT-PCR of *katG* | TCTCATCGATGACATTTTCC |
| QkatE-F | Sense primer for qRT-PCR of *katE* | CTCTCATCGATGACTTCCAT |
| QkatE-R | Antisense primer for qRT-PCR of *katE* | GGGACTCATATGTTTCGAAG |
| QgshB_F | Sense primer for qRT-PCR of *gshB* | TCGACATGGCCTATATCACC |
| QgshB_R | Antisense primer for qRT-PCR of *gshB* | CAGATCGGAGAATTCGGTGA |
| QaapJ_F | Sense primer for qRT-PCR of *aapJ* | ATTGCTGCGCCGGAGAGTTC |
| QaapJ_R | Antisense primer for qRT-PCR of *aapJ* | CTGACGCTTCCGGCAATTGG |
| QbraC_F | Sense primer for qRT-PCR of *braC* | TCTGTCGGCAGTGGCTCTGA |
| QbraC_R | Antisense primer for qRT-PCR of *braC* | AGTTGAAGTGGCCGATGACA |

*Restriction sites are underlined.

**Supplementary Table S2 Expression patterns of *gpxA* and *gshR* gene in symbiotic nodules.** Gene expression levels were examined by real-time RT-PCR. Nodules were collected on different days after inoculation with RL3841. Relative expression of *gpxA* and *gshR* genes in symbiotic nodule bacteroids compared with wild type RL3841 cells growth in AMS Glc/NH_4_^+^ medium. Data are the average of three independent biological samples (each with three technical replicates). ^a^ Superscript asterisk indicates no significant difference in relative expression.

| **Nodules** | **Average Expression** | |
| --- | --- | --- |
|  | ***gpxA*** | ***gshR*** |
| 14 days | 0.52±0.13^a^ | 1.48±0.32^a^ |
| 28 days | 0.88±0.17^a^ | 1.21±0.14^a^ |
| 42 days | 1.03±0.20^a^ | 0.89±0.19^a^ |

**
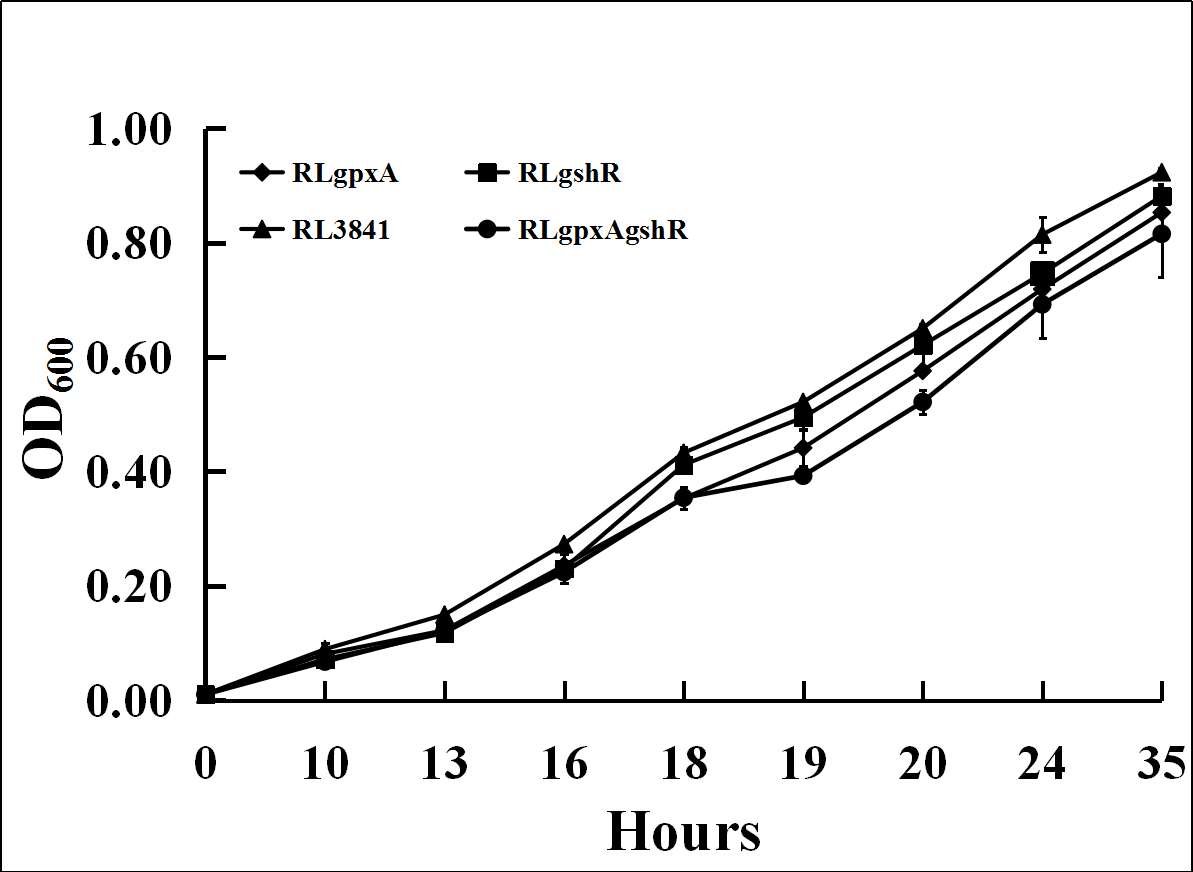
**

**Supplementary Figure S1 Growth of *R. leguminosarum* strains on AMS medium.**

Data are from three biological samples plus and minus the standard deviation (± SD).
